# PHGDH Orchestrates Cell Cycle Progression to Drive Cardiomyocyte Proliferation and Myocardial Regeneration via TGF‐β/Smad Signalling Pathway

**DOI:** 10.1111/cpr.70123

**Published:** 2025-09-10

**Authors:** Han Zhang, Li Zhang, Zehao Feng, Xing Li, Zhaohui Qiu, Xingyun Wang, Lingmei Qian

**Affiliations:** ^1^ Department of Cardiology & Hongqiao International Institute of Medicine Tongren Hospital, Shanghai Jiao Tong University School of Medicine Shanghai China; ^2^ Department of Cardiology Wuxi no. 2 People's Hospital (Jiangnan University Medical Center) Jiangsu Province China

**Keywords:** cardiomyocyte proliferation, cell cycle, myocardial regeneration, PHGDH

## Abstract

The mature mammalian heart has limited ability for self‐repair and regeneration. Here, we establish phosphoglycerate dehydrogenase (PHGDH) as a crucial key for cardiomyocyte proliferation, with diminishing expression during postnatal cardiac development. PHGDH overexpression promoted myocardial regeneration and cardiac function in apical resection‐operated mice, whereas inhibition by NCT‐503 inhibited these processes. In vitro, PHGDH stimulated the proliferation of cardiomyocytes (CMs), while NCT‐503 abolished its effect. Mechanistically, PHGDH activated the cell cycle and TGF‐β/Smad signalling. Moreover, PHGDH significantly enhances cardiac repair and stimulates cardiomyocyte proliferation in adult mice following myocardial infarction. Our study demonstrates that upregulating PHGDH promotes CM proliferation and myocardial regeneration, offering a promising therapeutic target for myocardial repair.

## Graphical abstract

PHGDH promoted cardiomyocyte proliferation and myocardial regeneration via cell cycle and TGF‐β/Smad signalling pathways during cardiac repair. Moreover, PHGDH benefits cardiac repair and cardiomyocyte proliferation in mammalian mice following MI.

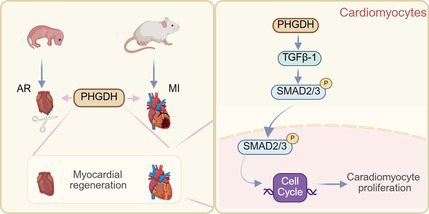

## 1 Introduction

Heart diseases remain a major global cause of death, primarily because of the limited regenerative capacity of the mammalian heart after myocardial injury [1]. Hearts in neonatal mice possess regenerative capacity within the first week after birth [2, 3, 4]; however, this capacity significantly decreases on postnatal day 7 (P7), coinciding with cell cycle arrest in cardiomyocytes (CMs) [5]. Lost CMs and injured myocardium were replenished at postnatal day 1 (P1) in a neonatal mice apical resection (AR) model; however, these effects were limited at P7 resection [6], providing robust evidence of the time window effect on cardiac regeneration. Gaining insight into the molecular mechanisms reactivating CM proliferation within a regenerative time window may provide potential approaches for treating injured mammalian hearts.

Phosphoglycerate dehydrogenase (PHGDH) is the initial and rate‐limiting enzyme for serine biosynthesis from the glycolytic intermediate 3‐phosphoglycerate [7, 8]. PHGDH exerts a significant direct influence on promoting tumour growth in multiple classifications of cancer, including breast cancer, colorectal cancer, and melanoma [9, 10, 11, 12]. Additionally, it is a metabolic checkpoint of macrophage polarisation and proliferation [13] it also upregulates the growth of vascular endothelial cells, which has been reported to be a consequence of intracellular serine biosynthesis [14]. However, the specific role of PHGDH in CMs remains unclear.

In this study, we establish PHGDH as crucial in CM proliferation, with diminishing expression during postnatal cardiac development. PHGDH promoted CM proliferation and myocardial regeneration in vivo and in vitro. Mechanistically, it activated CM proliferation via cell cycle and TGF‐β/Smad signalling during cardiac repair; blocking TGF‐β impaired this regenerative capacity. PHGDH benefits cardiac repair and CM proliferation in mammalian mice following MI. Together, this study demonstrated that PHGDH promotes the CMs proliferation and myocardial regeneration by modulating the cell cycle and TGF‐β/Smad signalling pathways. These findings suggest that manipulating PHGDH could be a potential therapeutic strategy for treating cardiac injuries.

## 2 Materials and Methods

### 2.1 Antibodies and Reagents

Antibodies against Ki67 (ab15580), Cardiac Troponin T (ab8295), and cyclin‐dependent kinase 2 (CDK2) (ab32147) were purchased from Abcam (Cambridge, United Kingdom). Antibodies against Cyclin D1 (#2978), Cyclin A2 (#81754), and β‐actin (#4967) were purchased from Cell Signalling Technology (USA). Antibodies against PHGDH (14719–1‐AP), Smad2 (12570–1‐AP), and TGF‐β1 (21898–1‐AP) were acquired from Proteintech, USA. Antibodies against phospho‐Smad3‐S423/S425 (AP0727) and phospho‐Smad2‐S467 (AP0269) were purchased from ABclonal Technology Co. Ltd. The antibody against Smad3 (AF1501) was purchased from Beyotime. NCT‐503 (HY‐101966), and SB‐431542 (HY‐10431) were purchased from MCE (Princeton, NJ, USA).

### 2.2 Animals

All animal experiments followed the guidelines of the Animal Care and Use Committee of Tongren Hospital (affiliated with Shanghai Jiao Tong University School of Medicine) and the Guide for the Care and Use of Laboratory Animals published by the National Institutes of Health (NIH Publications No. 85–23, revised 1996). Mice with an ICR background were obtained from Zhejiang Vital River Laboratory Animal Technology Co. Ltd. Age and sex‐matched animals were used.

### 2.3 AR Surgery

An AR surgery model was created with P1 neonatal ICR mice. Briefly, neonatal mice were anaesthetised on ice for nearly 3 min. After skin incision and intercostal muscle separation, the heart was softly extruded out from the 4th intercostal space, and 15% of the heart apex tissue was excised using iridectomy scissors. The chest and skin were sutured after resection, and mice were resuscitated under a heating lamp. The procedure was identical in sham mice except for AR. Once the heart apex muscle was resected, AAV9: cTNT‐PHGDH or AAV9: cTNT‐Ctrl were administered into the resected myocardium bordering zone from two locations (left and right) with 3 μL viral genomes for each point using a 36G needle microinjector. Each mouse was treated with 3 × 10^7^ plaque‐forming units of viral genome dissolved in 6 μL phosphate‐buffered saline (PBS). The AAV9: cTNT‐PHGDH and AAV9: cTNT‐Ctrl were constructed by and purchased from OBiO Technology (Shanghai) Corp Ltd.

### 2.4 Myocardial Infarction Model

An MI model was established using 8‐week‐old adult mice. Anaesthesia was induced with a 5% isoflurane‐O_2_ mixture and maintained at 2%. Mice were continuously supplied with 1.5% isoflurane‐O_2_ for stable respiration. Following skin incision and separation of the pectoral muscles, the heart was gently exteriorised through the third or fourth intercostal space. The left anterior descending (LAD) coronary artery was permanently ligated using 7–0 sutures. Following ligation, the thoracic cavity and skin were sealed with 4–0 sutures. Mice were then monitored postoperatively until full recovery. Sham‐operated controls underwent identical surgical procedures except for LAD ligation. Following LAD ligation, AAV9: cTNT‐PHGDH or AAV9: cTNT‐Ctrl were injected intramyocardially into the myocardium bordering the infarct zone at three sites.

### 2.5 Primary Neonatal Rat CM Isolation, Culture, and Adenoviral Infection

Ventricles from neonatal SD rats were used for CM isolation. After rapid decapitation and dissection, the ventricular myocardium was collected and sectioned into 1 mm^3^ heart tissue. The pieces were placed into 25 mL of digestion solution, including collagenase II (0.3 mg/mL; GIBCO, 2163320), and then shaken gently at 37°C for 20 min three times until the tissue was thoroughly digested. After each session, 5 mL of horse serum was added to terminate digestion. After centrifuging for 5 min at 1500 rpm, the supernatant was extracted and resuspended by adding media. The cells underwent 100‐μm filtration and were cultivated in high‐glucose Dulbecco's Modified Eagle Medium containing 5% fetal calf serum and 5% horse serum (37°C, 5% CO_2_) for 2 h to remove fibroblasts. The serum‐free medium was purchased from Gibco, USA, and is based on Dulbecco's Modified Eagle Medium, containing 4500 mg/L high glucose, L‐glutamine, and phenol red. CMs were then recollected and placed into 1% gelatin‐coated plastic at fitting density. The CM medium was changed after 24 h of culture before follow‐up experiments.

For adenovirus infection, CMs were cultured in a serine‐free medium for transfection to approximately 70% confluence before transfection with ADV: cTNT‐PHGDH or ADV: cTNT‐Ctrl (MOI = 50) for 48 h. Western blot was used to reveal the efficiency of ADV‐PHGDH infection. ADV: cTNT‐PHGDH and ADV: cTNT‐Ctrl were purchased from OBiO Technology (Shanghai) Corp Ltd.

### 2.6 Echocardiography

A Vevo‐2100 echocardiography system was used to measure cardiac function, with 2.5% isoflurane in air used to anaesthetise mice. The mice were kept on a heated platform under conditions ensuring spontaneous breathing. During the measuring process, heart rates were monitored within the range of 400 to 550 bpm. In the AR and MI model, short‐axis images were captured at the papillary muscles. The left ventricular contractile function parameters were examined by supporting software under M‐mode. All results were obtained at the end of diastole and systole across five cardiac cycles.

### 2.7 Histological Analysis

Hearts were washed with PBS and then fixed in 4% paraformaldehyde (PFA; Beyotime, Shanghai) at room temperature for 48 h. After dehydration with ethanol and xylene, the samples were paraffin‐embedded and sectioned frontally at 5‐μm thickness. The AR and MI model heart samples were constantly sectioned until the injured section was observed. Masson's Trichrome staining was performed based on a standard operating procedure.

### 2.8 Western Blot Analysis

Heart tissues and CMs were collected and immersed in radioimmunoprecipitation assay buffer (Beyotime, Shanghai) with 0.1% phenylmethanesulfonyl fluoride for extracting protein. After sufficient cleavage, all protein samples had sodium dodecyl sulfate loading buffer added before heating for protein denaturation. Nearly 20 μg of total protein was added for sodium dodecyl sulfate‐polyacrylamide gel electrophoresis before transferring to a 0.2‐μm PVDF membrane (Merck Millipore, Billerica, MA, USA). Following blocking, the membranes were incubated overnight with primary antibodies at 4°C. The membranes then underwent incubation with secondary antibodies. The immunoreactive signals were observed with a Tanon‐5200 analyser (Shanghai, China) using an Omni‐ECL Femto Light Chemiluminescence Kit (Shanghai Yamay Biomedical Technology Co. Ltd.).

### 2.9 Immunofluorescence

Cultured CMs were washed twice with PBS, fixed in 4% PFA for 15 min at room temperature, and permeabilised with 0.5% Triton X‐100 in PBS for 10 min. CMs were then incubated with primary antibodies at 4°C overnight, followed by fluorescently labelled secondary antibodies in the dark for 1 h. Nuclei were stained with DAPI for 10 min; images were captured using a fluorescence microscope and analysed with ImageJ software. For heart tissue samples, hearts were harvested, fixed in 4% PFA, and sectioned at 5 μm. Sections were immersed in citric acid buffer (pH 9.5), boiled in a microwave for 15 min, and blocked with 10% fetal calf serum for 1 h. Sections were incubated with primary antibodies at 4°C overnight and secondary antibodies for 1 h at room temperature. After DAPI staining, images were captured from three random fields per section and analysed using ImageJ.

### 2.10 RNA Sequencing

For RNA sequencing (RNA‐Seq) and data analysis, CMs were treated with NCT‐503 (10 μM) or Ctrl‐PBS for 48 h, and total RNA was retrieved using TRIzol extraction. The construction of sequencing libraries, as well as transcriptome sequencing and analysis, were conducted by OE Biotech Co. Ltd. (Shanghai, China). |log2FC| > 1.0 and *p* < 0.05 were used to analyse differentially expressed genes in DESeq2. R (v3.2.0). Subsequently, significantly enriched genes were determined in R (v 3.2.0) using hypergeometric distribution, Gene set enrichment analysis (GSEA), and the Kyoto Encyclopedia of Genes and Genomes (KEGG) pathway.

### 2.11 Statistical Analysis

The investigators were blinded when analysing cell, animal, and histological studies. Data from both sexes were included in the animal experiments. Data are presented as S.E.M. When comparing two groups, the unpaired Student's t‐test was applied. For comparisons among multiple groups, one‐way analysis of variance (ANOVA) was conducted. All statistical analyses were performed in GraphPad Prism (8.3.0), with *p* < 0.05 considered statistically significant.

## 3 Results

### 3.1 PHGDH Controls CM Proliferation

**FIGURE 1:**
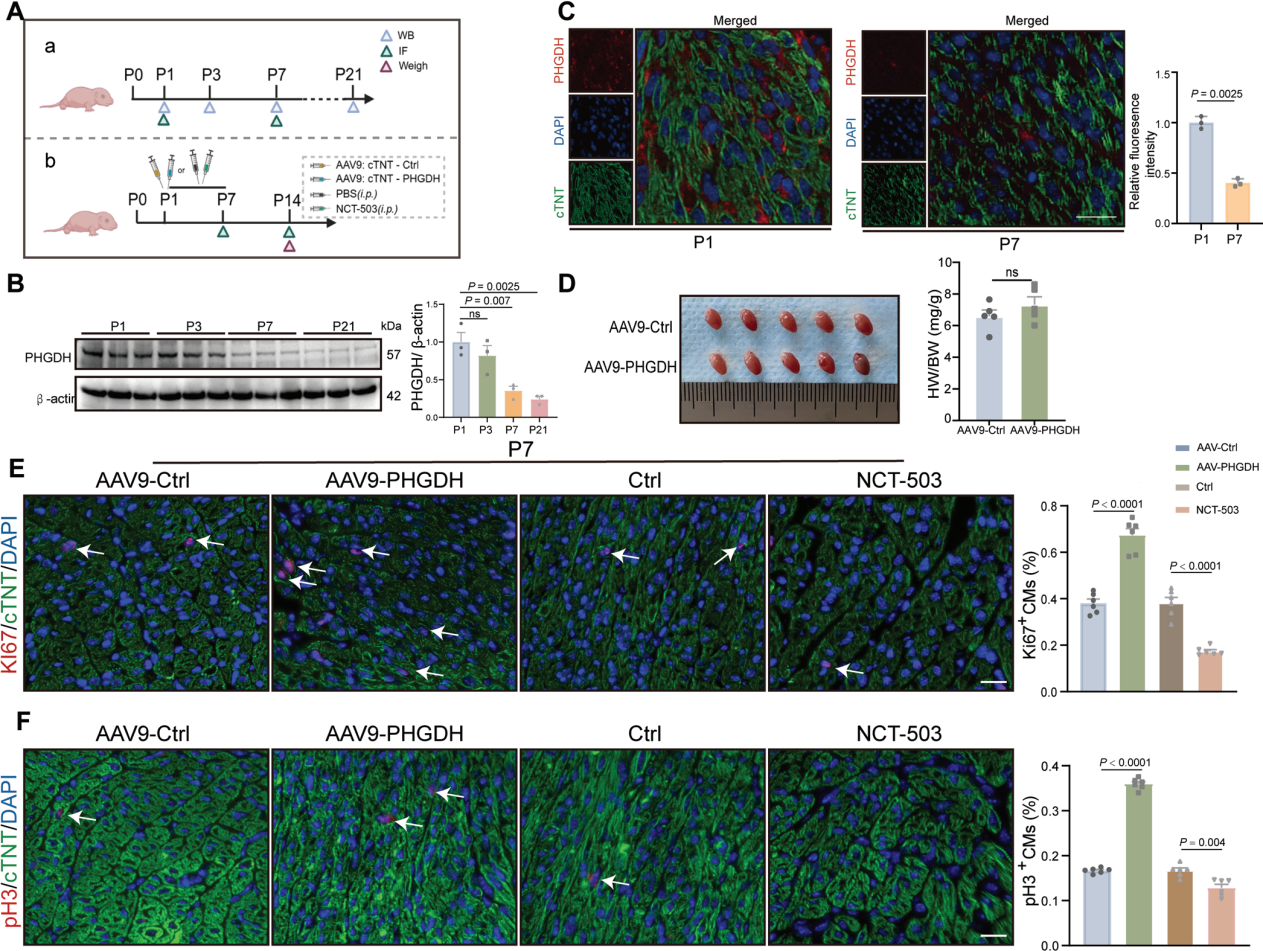
PHGDH is required for CM proliferation. (A) Experimental design: (a) Hearts from mice of different ages were collected for WB and IF. WB, Western Blot. IF, Immunofluorescence. (b) P1 mice were treated with cardiomyocyte (CM)‐specific AAV9: CTNT‐PHGDH or AAV9: CTNT‐Ctrl through intraperitoneal injection, while other P1 mice were continuously treated with NCT‐503 (40 mg/kg) or PBS for 7 d by intraperitoneal injection. (B) Western blot analysis and statistical evaluation of PHGDH expression in mice hearts at P1, P3, P7, and P21 (*n* = 6 per group). *P* value analysed by one‐way ANOVA. Data are displayed as mean ± S.E.M. (C) Representative immunostaining images of PHGDH expression in mice hearts at P1 and P7 (*n* = 6 per group). *P* value analysed by two‐tailed unpaired Student's t‐test. Data are displayed as mean ± S.E.M. (D) Cardiac morphology and HW/BW ratio between the AAV9: CTNT‐Ctrl and AAV9: CTNT‐PHGDH groups at P14 (*n* = 5/group). *P* value analysed by two‐tailed unpaired Student's t‐test. Data are displayed as mean ± S.E.M. (E and F) Representative immunostaining pictures and statistical analysis of Ki67^+^ or pH 3^+^ (red) CM numbers in mice hearts at P7 treated with AAV9: CTNT‐PHGDH or AAV9: CTNT‐Ctrl (*n* = 6 per group). Scale bars: 20 μm. *P* value assessed by one‐way ANOVA. Data are expressed as mean ± S.E.M.

We initially examined PHGDH expression in the heart throughout postnatal development to elucidate its role in CM proliferation. The protein level of PHGDH in postnatal mouse cardiac tissue progressively decreased over time, with a large reduction by P7 before remaining at a significantly low level until P21 (Figure 1A,B). In immunostaining of PHGDH expression in heart samples, expression was downregulated in P7 mice compared with P1 mice (Figure 1C). In line with a previous report on the regenerative time window, immunostaining was performed for proliferative markers Ki67^+^ and pH 3^+^ with anti‐cardiac troponin T (cTnT). There was a substantial decrease in the proliferative potential of P7 compared with P1 mouse hearts (Figure S1). These findings indicate that PHGDH downregulation coincides with the loss of CM proliferation during neonatal cardiac development, implying a potential role for PHGDH in cardiac regenerative capacity.

To uncover whether PHGDH is involved in CM proliferation, P1 mice were treated with CM‐specific AAV9: cTNT‐PHGDH or AAV9: cTNT‐Ctrl; the other group of P1 mice was administered PBS or the PHGDH inhibitor NCT‐503 (40 mg/kg) intraperitoneally for 7 d [15]. Heart samples were harvested at P7 and P14 for relevant trials. Significant upregulation in PHGDH of P7 mice treated with AAV9: cTNT‐PHGDH was seen compared with those treated with AAV9: cTNT‐Ctrl (Figure S2). The HW/BW ratio did not significantly differ between the two groups (Figure 1D). We further detected the potential of PHGDH to promote myocardial proliferation through immunofluorescence staining of proliferation markers with cTnT in P7 and P14 heart tissue, revealing a significant increase in the percentage of Ki67^+^ and pH 3^+^ CMs in P7 and P14 mice administered AAV9: cTNT‐PHGDH (Figure 1E,F and Figure S3). These results indicate that PHGDH increased neonatal CM proliferation.

### 3.2 PHGDH Promotes Myocardial Regeneration Following AR in Neonatal Mice

**FIGURE 2:**
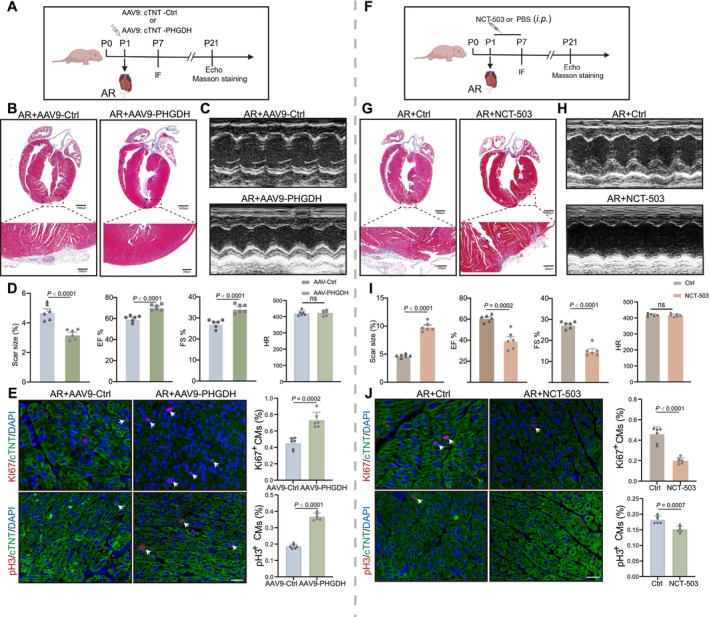
PHGDH promotes myocardial regeneration following apical resection (AR) in neonatal mice. (A) A diagram showing P1 mice were treated with AR and subsequently infected with CM‐specific AAV9: CTNT‐PHGDH or AAV9: CTNT‐Ctrl. Heart samples were harvested at 7 and 21 dpr for the indicated experiments. (B) Masson's trichrome staining for heart regeneration and scar size in mice at 21 dpr treated with CM (C‐specific AAV9: CTNT‐PHGDH) compared with AAV9: CTNT‐Ctrl (*n* = 6/group). Scale bars: 1000 μm. (C) Echocardiography images in mice 21 dpr treated with CM‐specific AAV9: CTNT‐PHGDH or AAV9: CTNT‐Ctrl. (D) Analysis of Masson staining, EF, FS, and HR in mice 21 dpr treated with CM‐specific AAV9: CTNT‐PHGDH relative to AAV9: CTNT‐Ctrl. *P* value compared with the AAV9‐Ctrl group assessed by two‐tailed unpaired Student's t‐test. Data are expressed as mean ± S.E.M. (E) Immunostaining images and quantitative analysis of Ki67^+^ or pH 3^+^ (red) CMs in mice hearts at P7 treated with AAV9: CTNT‐PHGDH or AAV9: CTNT‐Ctrl (*n* = 6/group). Scale bars: 20 μm. *P* value assessed by two‐tailed unpaired Student's t‐test. Data are expressed as mean ± S.E.M. (F) A diagram demonstrating that P1 mice were treated with AR and PBS or NCT‐503 (40 mg/kg). Heart samples were harvested at 7 and 21 dpr for the indicated experiments. Cardiac function was evaluated through echocardiography at 21 dpr. (G) Representative images of Masson staining of heart regeneration and scar size in mice 21 dpr treated with NCT‐503 relative to those treated with PBS (*n* = 6/group). Scale bars: 1000 μm. (H) Representative echocardiography images in mice treated with PBS or NCT‐503 at 21 dpr. (I) Analysis of Masson staining, EF, FS, and HR in mice treated with PBS or NCT‐503 at 21 dpr. *P* value compared with the PBS group as per a two‐tailed unpaired Student's t‐test. (J) Immunostaining images and statistical analysis of Ki67^+^ or pH 3^+^ (red) CMs in mice hearts at P7 treated with PBS or NCT‐503 (*n* = 6/group). Scale bars: 20 μm. *P* value assessed via two‐tailed unpaired Student's t‐test. Values are shown as mean ± S.E.M.

To further validate the potential involvement of PHGDH in myocardial regeneration after injury, we created an AR model in P1 mice and subsequently administered AAV9: cTNT‐PHGDH or AAV9: cTNT‐Ctrl; the other group was treated with PHGDH inhibitor NCT‐503 or PBS for 7 days consecutively via intraperitoneal injection (Figure 2A,F). Masson's staining analysis revealed lower fibrosis at 21 days post resection (dpr) in AAV9: cTNT‐PHGDH mice. Echocardiography demonstrated a substantial enhancement in the systolic function of the left ventricle in AAV9: cTNT‐PHGDH mice at 21 dpr compared with the AAV9: cTNT‐Ctrl group through ejection fraction (EF) and fraction shortening (FS) increase (Figure 2B–D). However, NCT‐503 treatment inhibited heart function recovery and resulted in more myocardial scars than in the PBS group (Figure 2G–I). Additionally, we created an AR model in P7 mice; Masson staining showed a decreased fibrotic scar size at 21 dpr in the PHGDH group compared to the Ctrl group (Figure S4). This suggests that PHGDH is necessary for myocardial regeneration.

To determine whether PHGDH stimulates CM proliferation in injured hearts, immunostaining was performed for the proliferative markers Ki67^+^ and pH 3^+^ at 7 dpr. The proportion of pH 3^+^ and Ki67^+^ CMs in P7 AAV9: cTNT‐PHGDH mice was elevated, while NCT‐503 treatment inhibited this effect (Figure 2E,J). These observations indicate that PHGDH increases the capacity for CM proliferation and neonatal myocardial regeneration after cardiac injury.

### 3.3 PHGDH Promotes Primary Neonatal Rat CM Proliferation In Vitro

**FIGURE 3:**
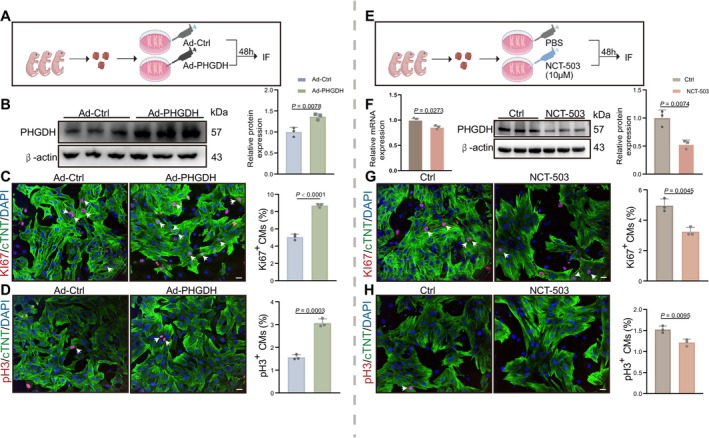
PHGDH promotes primary neonatal rat CM proliferation in vitro. (A) A schematic diagram depicting the experimental design for Figure 3B–D. (B) Western blot analysis and statistical analysis of PHGDH protein expression in Ad‐PHDGH‐treated. CMs compared with the Ad‐Ctrl group (*n* = 3/group). (C and D) Immunostaining images and statistical analysis of Ki67^+^ or pH 3^+^ (red) CMs infected with Ad: CTNT‐PHGDH or Ad: CTNT‐Ctrl (*n* = 3/group). Scale bars: 20 μm. (E) A schematic diagram depicting the experimental design for Figure 3F–H. (F) The statistical analysis of PHGDH mRNA and protein expression in 10 μM NCT‐503‐treated CMs for 48 h compared with the PBS‐treated group (*n* = 3/group). (G and H) Immunostaining images and statistical analysis of Ki67^+^ or pH 3^+^ (red) CMs treated with PBS or NCT‐503 (*n* = 3/group). Scale bars: 20 μm. *P* value assessed by two‐tailed unpaired Student's t‐test. Data are expressed as mean ± S.E.M.

Considering that PHGDH modulation significantly influenced myocardial regeneration in vivo, we sought to establish if PHGDH affected CM proliferation. We extracted neonatal rat CMs and cultivated them in a medium without serine before administering Ad: cTNT‐PHGDH or Ad: cTNT‐Ctrl for 48 h for overexpression experiments; the other group was treated with 10 μM NCT‐503 or PBS for 48 h (Figure 3A,E). Ad: cTNT‐PHGDH was effectively overexpressed in mice CMs, and NCT‐503 inhibited PHGDH mRNA and protein expression (Figure 3B,F). Although the established mechanism of NCT‐503 is as an enzyme activity inhibitor, we carried out qPCR and found that NCT‐503 lowers PHGDH mRNA level, indicating that the compound suppresses transcription. To establish whether PHGDH stimulates CM proliferation, immunostaining was performed for the proliferative markers of Ki67^+^ and pH 3^+^ following infection with Ad: cTNT‐PHGDH or Ad: cTNT‐Ctrl. Ki67^+^ and pH 3^+^ increased in the PHGDH group (Figure 3C,D). Additionally, the proportions of Ki67^+^ and pH 3^+^ were significantly downregulated in neonatal rat CMs treated with NCT‐503 (Figure 3G,H). We transfected CMs with siPHGDH and observed a decrease in Ki67 and pH 3 (Figure S5). These findings indicate that PHGDH plays a regulatory role in CM proliferation.

### 3.4 PHGDH Stimulates CM Proliferation Through Cell Cycle and TGF‐β1/Smad2/3 Signalling Activation

**FIGURE 4:**
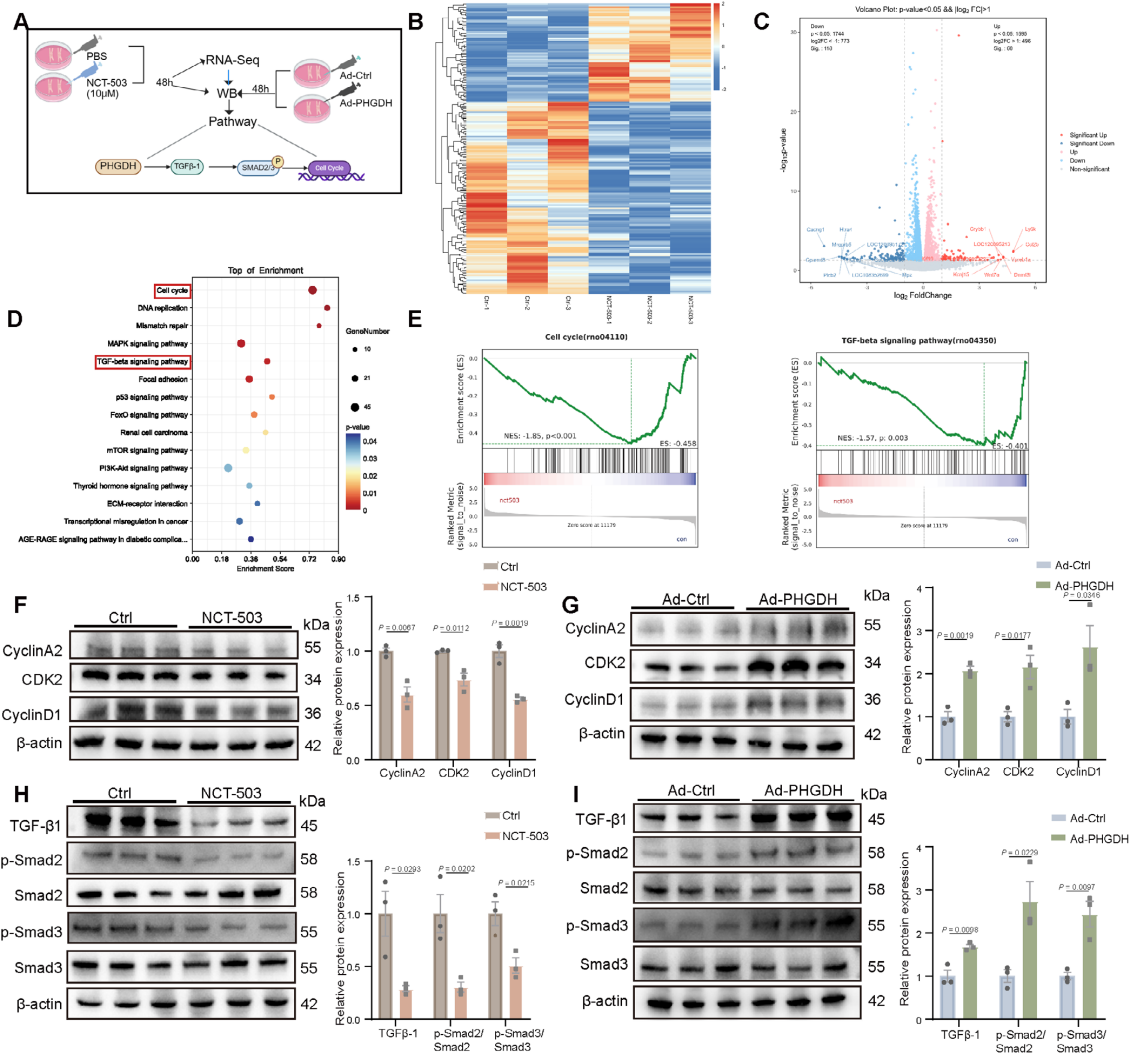
PHGDH stimulates CM proliferation by activating the cell cycle and TGF‐β/Smad signalling pathways. (A) A diagram outlining the experimental setup for Figure 4B–I, RNA‐Seq analyses from CMs treated with PBS or 10 μM NCT‐503 for 48 h (*n* = 3/group). (B and C) Heatmap and volcano plot showing differentially expressed genes in CMs treated with PBS or 10 μM NCT‐503 for 48 h (|log2FC| > 1.0, *p* < 0.05). (D) KEGG analysis illustrating the top overrepresented terms for downregulated genes (|log2FC| > 1.0, *p* < 0.05) ranked by significance. (E) GSEA showing that the cell cycle and TGF‐β signalling were downregulated following NCT‐503 treatment. (F) The expression and statistical analysis of CyclinA2, CDK2, CyclinD1 in CMs treated with PBS or NCT‐503 as determined by western blot (*n* = 3/group). (G) The expression and statistical analysis of CyclinA2, CDK2, CyclinD1 in CMs transfected with Ad: CTNT‐PHGDH or Ad: CTNT‐Ctrl (*n* = 3/group). (H) Western blot images and statistical analysis of protein expression of TGF‐β, p‐Smad2/Smad2, p‐Smad3/Smad3, and β‐actin in CMs treated with PBS or NCT‐503 (*n* = 3/group). (I) Western blot images and statistical analysis of protein expression of TGF‐β, p‐Smad2/Smad2, p‐Smad3/Smad3, and β‐actin in CMs treated with Ad‐Ctrl and Ad‐PHGDH (*n* = 3/group). *P* values assessed by two‐tailed unpaired Student's t‐test. Results are shown as mean ± S.E.M.

The aforementioned findings indicate the role of PHGDH in promoting proliferation in neonatal mice hearts and CMs. To investigate downstream signalling involved in PHGDH induction of CM proliferation, RNA‐Seq was carried out to analyse primary neonatal rat CM gene expression profiles with the administration of PHGDH inhibitor NCT‐503 for 48 h (Figure 4A). The heatmap and volcano plot showed differentially expressed genes in CMs treated with NCT‐503 compared with PBS (Figure 4B,C). KEGG analysis indicated that TGF‐β signalling and cell cycle were enriched by assessing downregulated genes after NCT‐503 treatment (Figure 4D). GSEA revealed that cell cycle (rno041110) and TGF‐β signalling (rno04350) were downregulated with NCT‐503 treatment (Figure 4E).

To confirm the RNA‐Seq results, we identified some classical cell cycle markers (Cyclin A2, CDK2, and Cyclin D1) using western blot in NCT‐503 or PBS‐treated CM samples. Their expression was markedly lower in the NCT‐503 group (Figure 4F). To directly assess the effects of PHGDH on CMs, we also examined the above protein markers after infecting Ad: cTNT‐PHGDH, where they were significantly increased (Figure 4G). We further measured TGF‐β1, Smad2, and Smad3 activation in CMs by administering NCT‐503 or PBS and Ad: cTNT‐PHGDH or Ad: cTNT‐Ctrl. TGF‐β1; phosphorylated Smad2/3 expression was downregulated with NCT‐503 treatment but upregulated with Ad: cTNT‐PHGDH infection (Figure 4H,I). These data show that PHGDH stimulates CM proliferation by activating cell cycling and TGF‐β1/Smad2/3 signalling.

### 3.5 PHGDH Promotes Injured Myocardial Regeneration by Activating Cell Cycle and TGF‐β1/Smad2/3 Signalling

**FIGURE 5:**
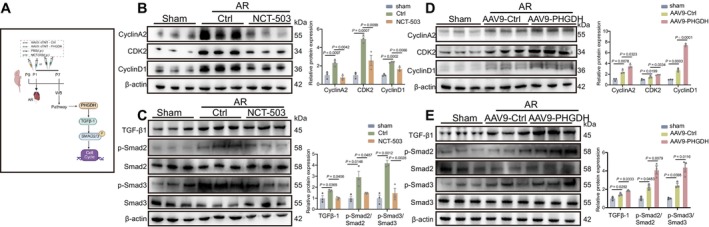
PHGDH promotes myocardial regeneration after injury by activating the cell cycle and TGF‐β/Smad signalling. (A) A schematic diagram depicting the experimental layout for Figure 5B–E. (B) The expression and statistical analysis of PHGDH, CyclinA2, CDK2, CyclinD1 in mice hearts of the P7 sham and 7 dpr groups treated with PBS or NCT‐503 (40 mg/kg) as determined by western blot (*n* = 6/group). (C) Western blot and statistical analysis of the expression of TGF‐β, p‐Smad2/Smad2, p‐Smad3/Smad3, and β‐actin proteins in mice hearts of the P7 sham and 7 dpr groups treated with PBS or 40 mg/kg NCT‐503 (*n* = 6/group). (D) The expression and statistical analysis of PHGDH, CyclinA2, CDK2, CyclinD1 in mice hearts of the P7 sham and 7 dpr groups transfected with AAV9: CTNT‐PHGDH or AAV9: CTNT‐Ctrl, as determined by western blot (*n* = 6/group). (E) Western blot and statistical analysis of expression of TGF‐β, p‐Smad2/Smad2, p‐Smad3/Smad3, and β‐actin proteins in mice hearts of the P7 sham and 7 dpr groups transfected with AAV9: CTNT‐PHGDH or AAV9: CTNT‐Ctrl (*n* = 6/group). *P* values assessed by one‐way ANOVA. Data are expressed as mean ± S.E.M.

Because PHGDH affects cell cycling and TGF‐β1/Smad2/3 signalling to influence cardiac regeneration in vitro, we then validated whether it regulates myocardial regeneration by targeting these pathways in vivo. We conducted AR or sham operations in P1 mice treated with NCT‐503 or PBS and AAV9: cTNT‐PHGDH or AAV9: cTNT‐Ctrl (Figure 5A). Cyclin A2, CDK2, and Cyclin D1 expression substantially improved after treatment with AAV9: cTNT‐PHGDH (Figure 5D) but decreased with NCT‐503 in mice hearts at 7 dpr (Figure 5B). To assess the relationship of PHGDH‐mediated TGF‐β signalling, we also extracted protein from 7 dpr and sham group mice hearts to measure the expression of TGF‐β1, p‐Smad2/Smad2, and p‐Smad3/Smad3. All increased 7 dpr in mice hearts treated with AAV9: cTNT‐PHGDH (Figure 5E), whereas they decreased in NCT‐503‐treated mice (Figure 5C). These results suggest that PHGDH promotes myocardial regeneration in neonatal mice after cardiac injury through cell cycle and TGF‐β1/Smad2/3 signalling.

### 3.6 TGF‐β Inhibitor Limits the Effect of PHGDH on CM Proliferation and Myocardial Regeneration in Neonatal Mice

**FIGURE 6:**
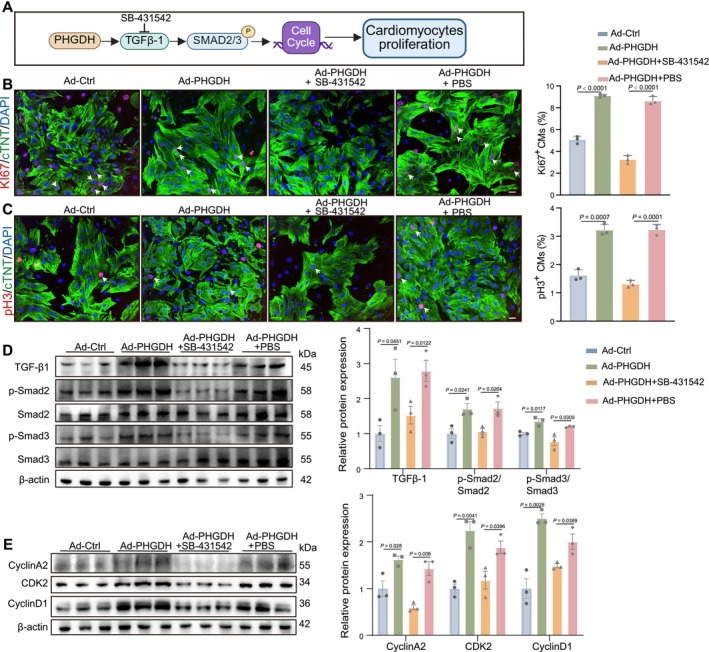
TGF‐β inhibitor inhibits the effect of PHGDH on CM proliferation. (A) schematic diagram depicting the experimental layout for Figure 6B–E. (B and C) Immunostaining images and statistical analysis of Ki67^+^ or pH 3^+^ (red) CMs treated with Ad: CTNT‐Ctrl, Ad: CTNT‐PHGDH, Ad: CTNT‐PHGDH+10 μM SB‐431542, or Ad: CTNT‐PHGDH+PBS (*n* = 3/group). Scale bars: 20 μm. (D and E) Western blot and statistical analysis of the expression of TGF‐β, p‐Smad2/Smad2, p‐Smad3/Smad3, CyclinA2, CDK2 and CyclinD1 in CMs treated with Ad: CTNT‐Ctrl, Ad: CTNT‐PHGDH, Ad: CTNT‐PHGDH +10 μM SB‐431542, or Ad: CTNT‐PHGDH + PBS (*n* = 3/group). *P* values assessed by one‐way ANOVA. Data are presented as mean ± S.E.M.

To further confirm whether cell cycle and TGF‐β1/Smad2/3 signalling mediate PHGDH‐induced CM proliferation, CMs were treated with TGF‐β inhibitor (SB‐431542) in the presence of Ad: cTNT‐PHGDH [16] (Figure 6A). The immunofluorescence of pH 3^+^ and Ki67^+^ showed that CM proliferation was limited with co‐treatment of SB‐431542 and PHGDH compared with the PHGDH group (Figure 6B,C). Cell cycle markers, TGF‐β1, and p‐Smad2/3 were increased in Ad: cTNT‐PHGDH‐treated CMs; however, this decreased with the addition of SB‐431542 (Figure 6D,E). Additionally, we treated CMs with TGF‐β1‐containing culture medium and observed a significant increase in the expression of cell cycle markers and the proportions of pH 3^+^ in neonatal rat CMs (Figure S6). The results suggested that TGF‐β inhibition hindered the potential of PHGDH to promote CM proliferation and myocardial regeneration, indicating that TGF‐β signalling was upstream of cell cycle activation in this model.

**FIGURE 7:**
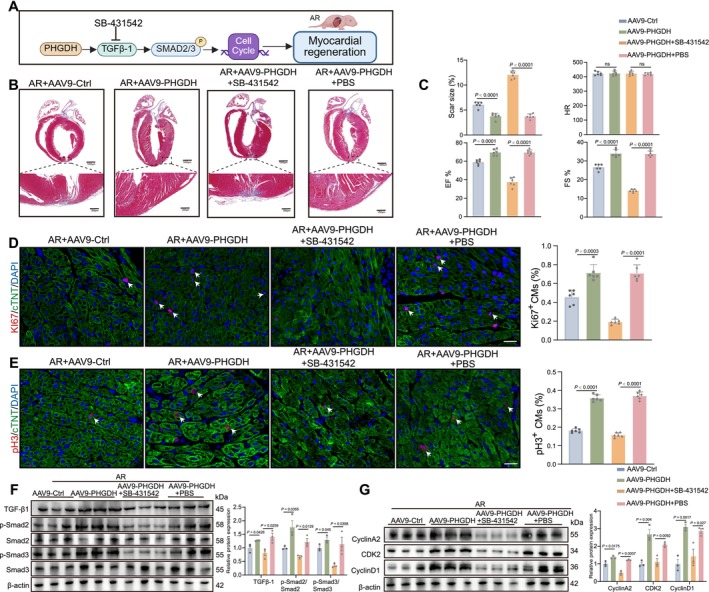
TGF‐β inhibitor limits the effect of PHGDH on myocardial regeneration in neonatal mice. (A) A schematic diagram depicting the experimental layout for Figure 7B–G. (B) Masson staining and scar size in mice 21 dpr treated with AAV9: CTNT‐Ctrl, AAV9: CTNT‐PHGDH, AAV9: CTNT‐PHGDH +10 mg/kg SB‐431542, or AAV9: CTNT‐PHGDH + PBS (*n* = 6/group). Scale bars: 1000 μm. (C) Analysis of Masson staining, EF, FS, and HR in mice 21 dpr treated with AAV9: CTNT‐Ctrl, AAV9: CTNT‐PHGDH, AAV9: CTNT‐PHGDH +10 mg/kg SB‐431542, or AAV9: CTNT‐PHGDH + PBS (*n* = 6/group). (D and E) Immunostaining images and statistical analysis of Ki67^+^ or pH 3^+^ (red) CMs in mice 7 dpr hearts treated with AAV9: CTNT‐Ctrl, AAV9: CTNT‐PHGDH, AAV9: CTNT‐PHGDH +10 mg/kg SB‐431542, or AAV9: CTNT‐PHGDH + PBS (*n* = 6/group). Scale bars: 20 μm. (F and G) Western blot and statistical analysis of the expression of TGF‐β, p‐Smad2/Smad2, p‐Smad3/Smad3, CyclinA2, CDK2, and CyclinD1 in mice hearts 7 dpr treated with AAV9: CTNT‐Ctrl, AAV9: CTNT‐PHGDH, AAV9: CTNT‐PHGDH +10 mg/kg SB‐431542, or AAV9: CTNT‐PHGDH + PBS as determined by western blot (*n* = 6/group). *P* value assessed by one‐way ANOVA. Data are presented as mean ± S.E.M.

Similarly, we treated neonatal mice with TGF‐β inhibitor (SB‐431542) continuously for 7 d after AR and injected AAV9: cTNT‐PHGDH simultaneously (Figure 7A). Cardiac function recovery was suppressed, and the fibrosis area was larger after PHGDH and SB‐431542 co‐treatment in mice hearts 21 dpr compared with PHGDH and PBS treatment (Figure 7B,C, and Figure S7). The capacity of CM proliferation was decreased in mice treated with SB‐431542 and PHGDH in mice heart immunostaining 7 dpr (Figure 7D,E). The expression levels of cell cycle markers, TGF‐β1, and p‐Smad2/3 were upregulated with PHGDH treatment but downregulated by co‐treatment with SB‐431542 at 7 dpr (Figure 7F,G). These data indicate that PHGDH‐induced CM proliferation and myocardial regeneration are mediated by cell cycle and TGF‐β1/Smad2/3 signalling.

### 3.7 PHGDH Enhances Myocardial Regeneration and CM Proliferation in Adult Mice After MI

**FIGURE 8:**
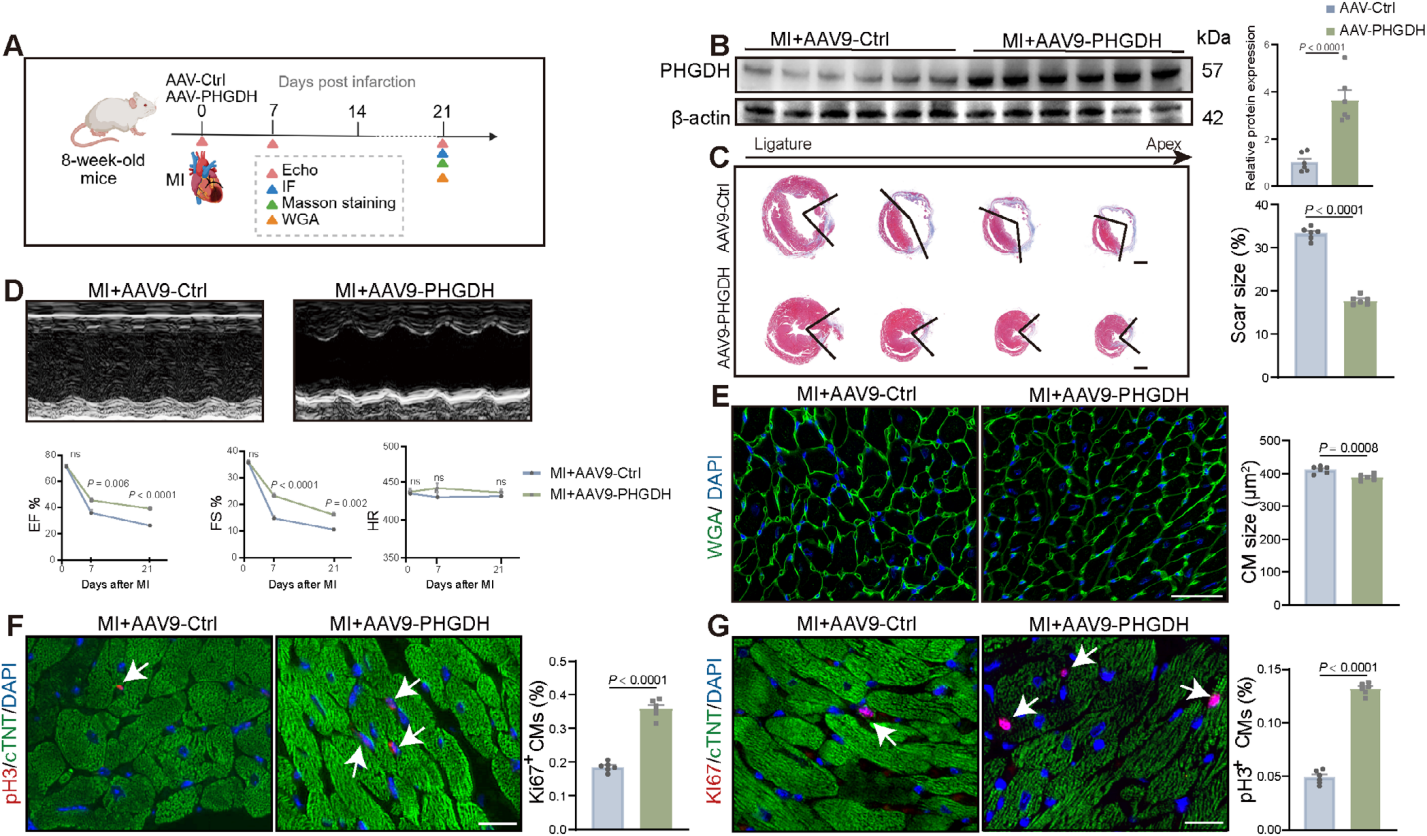
PHGDH enhances myocardial repair and CM proliferation in adult mice following MI. (A) Diagram showing 8‐week‐old mice treated with MI and subsequently injected intramyocardially with CM‐specific AAV9: CTNT‐PHGDH or AAV9: CTNT‐Ctrl. Echocardiography was performed at day 0 as well as 7 d after MI (dpi) and 21 dpi. Heart samples were collected at 7 and 21 dpi. (B) Western blot analysis and statistical analysis of PHGDH protein expression in AAV9: CTNT‐PHGDH‐treated 8‐week‐old mice with MI compared with the AAV9: CTNT‐Ctrl group (*n* = 6/group). (C) Masson staining and statistical analysis in mice 21 dpi treated with CM‐specific AAV9: CTNT‐PHGDH compared with those treated with AAV9: CTNT‐Ctrl (*n* = 6/group). Scale bars: 1000 μm. (D) Echocardiography images in mice 21 dpi and analysis of EF and FS in mice treated with CM‐specific AAV9: CTNT‐PHGDH or AAV9: CTNT‐Ctrl. (E) Quantification of CM size by wheat germ agglutinin (WGA) staining in AAV9: CTNT‐PHGDH or AAV9: CTNT‐Ctrl‐injected hearts at 21 dpi (*n* = 6/group). Scale bars: 20 μm. WGA (green), DAPI (blue). (F and G) Immunostaining images and statistical analysis of Ki67^+^ or pH 3^+^ (red) CMs in mice hearts at 7 dpi after treatment with AAV9: CTNT‐PHGDH or AAV9: CTNT‐Ctrl (*n* = 6/group). Scale bars: 20 μm. *P* value assessed by two‐tailed unpaired Student's t‐test. Data are presented as mean ± S.E.M.

To explore the role of PHGDH in adult animals, we utilised an MI model and intramyocardially injected AAV9: cTNT‐PHGDH or AAV9: cTNT‐Ctrl into 8‐week‐old mice (Figure 8A). Western blot was used to verify the overexpression of PHGDH (Figure 8B). There was lower fibrosis at 21 dpi in AAV9: cTNT‐PHGDH mice (Figure 8C), and the EF% and FS% were significantly improved at 21 dpi (Figure 8D). A significant reduction in CM size was seen at 21 d post‐MI (Figure 8E). Immunofluorescence staining also revealed a higher proportion of pH 3^+^ and Ki67^+^ CMs in 7 dpi AAV9: cTNT‐PHGDH‐treated mice (Figure 8F,G and Figure S8). These observations indicate that PHGDH enhances myocardial regeneration and CM proliferation in adult mice after MI.

## 4 Discussion

Enhancing CM proliferation is a highly promising approach for facilitating heart regeneration after cardiac injury [17, 18, 19]. We identified PHGDH as a crucial regulator of CM proliferation, with its expression decreasing during postnatal cardiac development. PHGDH overexpression promoted myocardial regeneration and cardiac function in AR mice, whereas PHGDH inhibition by NCT‐503 inhibited these effects. In vitro, it stimulated CM proliferation, while NCT‐503 abolished this. Mechanistically, PHGDH activated cell cycle and TGF‐β1/Smad2/3 signalling to promote CM proliferation. PHGDH also enhanced myocardial repair and CM proliferation in adult mice following MI.

In the mammalian heart, CM metabolism shifts from anaerobic glycolysis to mitochondrial phosphorylation after birth, simultaneously with cell‐cycle arrest [20]. Several studies have primarily focused on glycolysis and fatty acid oxidation in relation to CM proliferation [21, 22, 23]. Elevating glucose metabolism and inhibiting fatty acid oxidation enhances CM proliferation. However, the role of amino acid metabolism, particularly serine metabolism, in CM proliferation and myocardial regeneration has been less studied.

Our study identified PHGDH, a key enzyme in serine biosynthesis, as a crucial regulator of CM proliferation. PHGDH is a key regulator of a diverse array of biological processes, including promoting cell growth, regulating cellular senescence, and modulating metabolic reprogramming [24, 25]. It regulates endothelial cellular senescence and ageing through the PHGDH‐PKM2‐H3pT11 signalling axis [14]. Additionally, it exerts a pro‐proliferative effect on cultured cancer cells and osteoclasts [7]. In this study, we demonstrated the novel role of PHGDH in promoting CM proliferation and facilitating heart regeneration. PHGDH overexpression through heart‐specific adeno‐associated viruses promoted CM proliferation and myocardial regeneration in both AR neonatal mice and adult mice with MI.

The PHGDH level gradually decreased in postnatal mouse cardiac tissue, with a significant reduction by P7, coinciding with the loss of CM proliferation during neonatal cardiac development. The postnatal regeneration time window in neonatal mice hearts is when CMs have concurrently exited the cell cycle [26]. During cardiac development after birth, the cell cycle of CMs gradually stalls, with a reduction in the expression of mitogenic molecules, including cyclin and cyclin‐dependent kinases. However, several small molecules and proteins, such as LRP6, CNBP, and PKM2, stimulate quiescent CMs to undergo cytokinesis, activating them to re‐enter the cell cycle for regeneration [27, 28, 29]. Consistent with those studies, PHGDH promoted CM proliferation by promoting cell cycle re‐entry, with an increased proportion of Ki67 and pH 3‐positive CMs. Additionally, cell cycling was enriched in RNA‐Seq and further showed that PHGDH could upregulate the expression of Cyclin A2, CDK2, and Cyclin D1. This suggests that PHGDH could effectively trigger CMs to re‐enter cell cycling in vivo and in vitro.

Using RNA‐Seq, TGF‐β signalling was significantly down‐regulated with PHGDH inhibitor treatment. TGF‐β expresses two types of TGF‐β1 receptors: ALK‐1, which activates Smad1, 5, and 8; and ALK‐5, which activates Smad2 and 3 [30]. As a remodelling factor of regeneration, TGF‐β1 is linked to TGF‐β1/Smad2/3 signalling, which may promote angiogenesis, wound healing, axon growth, and anti‐apoptosis [31, 32]. Although investigations have discovered that activation of TGF‐β signalling causes heart failure and cardiac fibrosis in adult zebrafish [33], other researchers have reported that it can promote adult zebrafish heart valve regeneration by stimulating progenitor cell proliferation and promoting valve cell differentiation [34]. Additionally, whereas the TGF‐β signalling pathway causes cell arrest in various cell types, it also promotes the proliferation of endothelial and different mesenchymal cells under certain conditions [35, 36]. During CM proliferation and myocardial regeneration in neonatal mice, PHGDH induced TGF‐β1 activation and downstream Smad2/3 signalling in vivo and in vitro, while PHGDH inhibition by NCT‐503 inhibited them.

In addition to identifying PHGDH as a driver of cell cycle and TGF β1/Smad2/3 signalling, rescue experiments blocking the TGF‐β receptor with SB431542 were performed, inhibiting its effect on CM proliferation and myocardial regeneration. Additionally, cell cycle marker protein levels were decreased, indicating that cell cycling correlates with TGF‐β/Smad signalling. We treated CMs with TGF‐β and observed a significant increase in cell cycle marker expression, strengthening the evidence that TGF‐β signalling is upstream of cell cycle activation in this model. Although the TGF‐β receptor has a debatable role in adult heart injury and repair, TGF‐β promoted the proliferation of CMs in neonatal mice, potentially because of maturation features and heterogeneity of the myocardium in neonatal and adult mice. Targeting PHGDH promotes CM proliferation and myocardial regeneration through activating cell cycle and TGF‐β1/Smad2/3 signalling in neonatal mice.

In summary, our study highlights that PHGDH promoted CM proliferation and myocardial regeneration through detecting common proliferative markers (Ki67 and pH 3), cardiac function, and histological analysis. Considering that time and space can interfere with the accuracy of detecting proliferative markers, future investigations should employ more markers and advanced methodologies such as MADM or live‐cell imaging techniques to directly track CM division and validate proliferative activity. Mechanistically, PHGDH promoted CM proliferation and myocardial regeneration by activating cell cycle and TGF‐β1/Smad2/3 signalling in neonatal mice. In RNA‐Seq analysis, other pathways such as MAPK were also significantly enriched, requiring further exploration. PHGDH was also crucial for myocardial regeneration and cardiac function recovery in adult mice with MI. These results suggest that targeting PHGDH may represent a hopeful therapeutic strategy for the treatment of MI and heart failure.

## 5 Conclusion

Our findings clarify that PHGDH mediates CM proliferation and myocardial regeneration by activating the cell cycle and TGF‐β1/Smad2/3 signalling. To our knowledge, this study appears to be the first to investigate the effects of PHGDH on myocardial regeneration, indicating that targeting PHGDH could hold potential value for repairing myocardial injury.

## Author Contributions

Conceptualization: Xingyun Wang and Lingmei Qian. Methodology: Li Zhang, Han Zhang and Zehao Feng. Project administration: Han Zhang and Li Zhang. Supervision: Zhaohui Qiu and Lingmei Qian. Writing – original draft: Han Zhang. Writing – review and editing: Xingyun Wang and Lingmei Qian. Funding: Lingmei Qian. All authors have read and agreed to the published version of the manuscript.

## Ethics Statement

All animal experiments were approved by the ethics committee of Tongren hospital, Shanghai Jiao Tong University School of Medicine (committee reference number: A2023‐088‐01).

## Consent

All authors gave approval to publish the present study.

## Conflicts of Interest

The authors declare no conflicts of interest.

## Supporting information


**Data S1:** cpr70123‐sup‐0001‐Figures.docx.

**Data S2:** cpr70123‐sup‐0002‐supinfo.xlsx.

## Data Availability

The data that support the findings of this study are available on request from the corresponding author. The data are not publicly available due to privacy or ethical restrictions.
