# Supplementary material for: PHGDH Orchestrates Cell Cycle Progression to Drive Cardiomyocyte Proliferation and Myocardial Regeneration via TGF‐β/Smad Signalling Pathway
Source: Cell Prolif. 2025 Sep 10;59(4):e70123. doi: 10.1111/cpr.70123 (PMC13052272; doi:10.1111/cpr.70123)
Supplement: Supplementary file 1 — Data S1: cpr70123‐sup‐0001‐Figures.docx. [file CPR-59-e70123-s001.docx]

**Supporting Information**

**Title:** **PHGDH Orchestrates Cell Cycle Progression to Drive Cardiomyocyte Proliferation and Myocardial Regeneration via TGF-β/Smad Signalling Pathway**

**Running title: PHGDH Drives Adult Cardiomyocyte Proliferation**


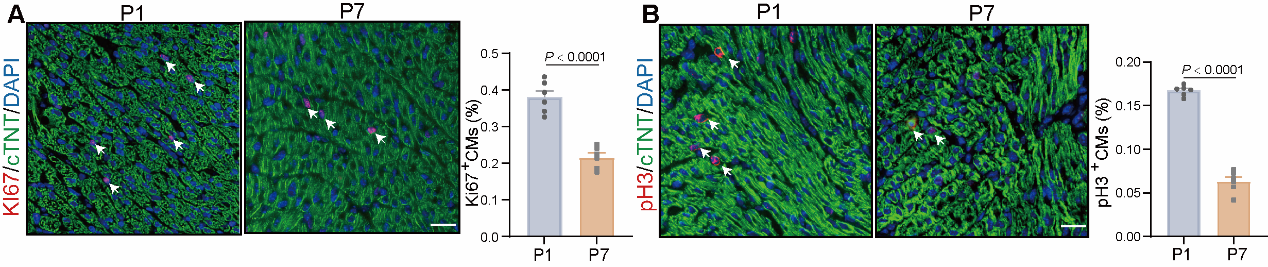


**Figure S1.**

(A and B) Immunostaining images and statistical analysis of Ki67^+^ or pH3^+^ (red) cardiomyocyte numbers in mice hearts at P1 and P7 (n=6/group). Scale bars, 20μm. *P* value analyzed by two-tailed unpaired Student’s t-test analysis. The data is displayed as mean ± S.E.M


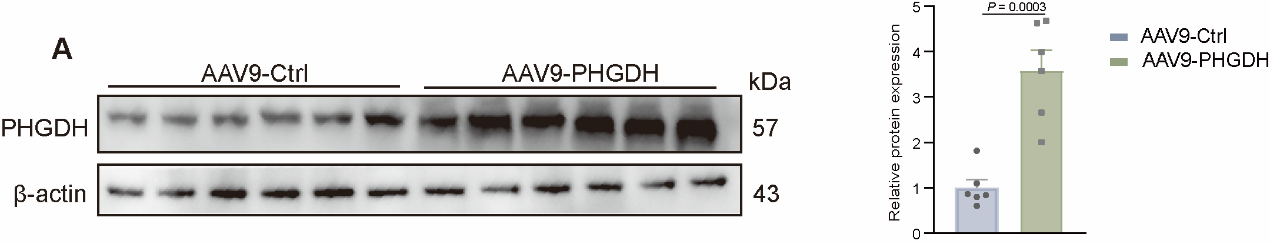


**Figure S2.**

(A) Western blot analysis and statistical analysis of PHGDH protein expression in AAV-PHDGH treated mice hearts compared with AAV-Ctrl group. (n= 6/group). *P* value analyzed by two-tailed unpaired Student’s t-test analysis. The data is displayed as mean ± S.E.M.


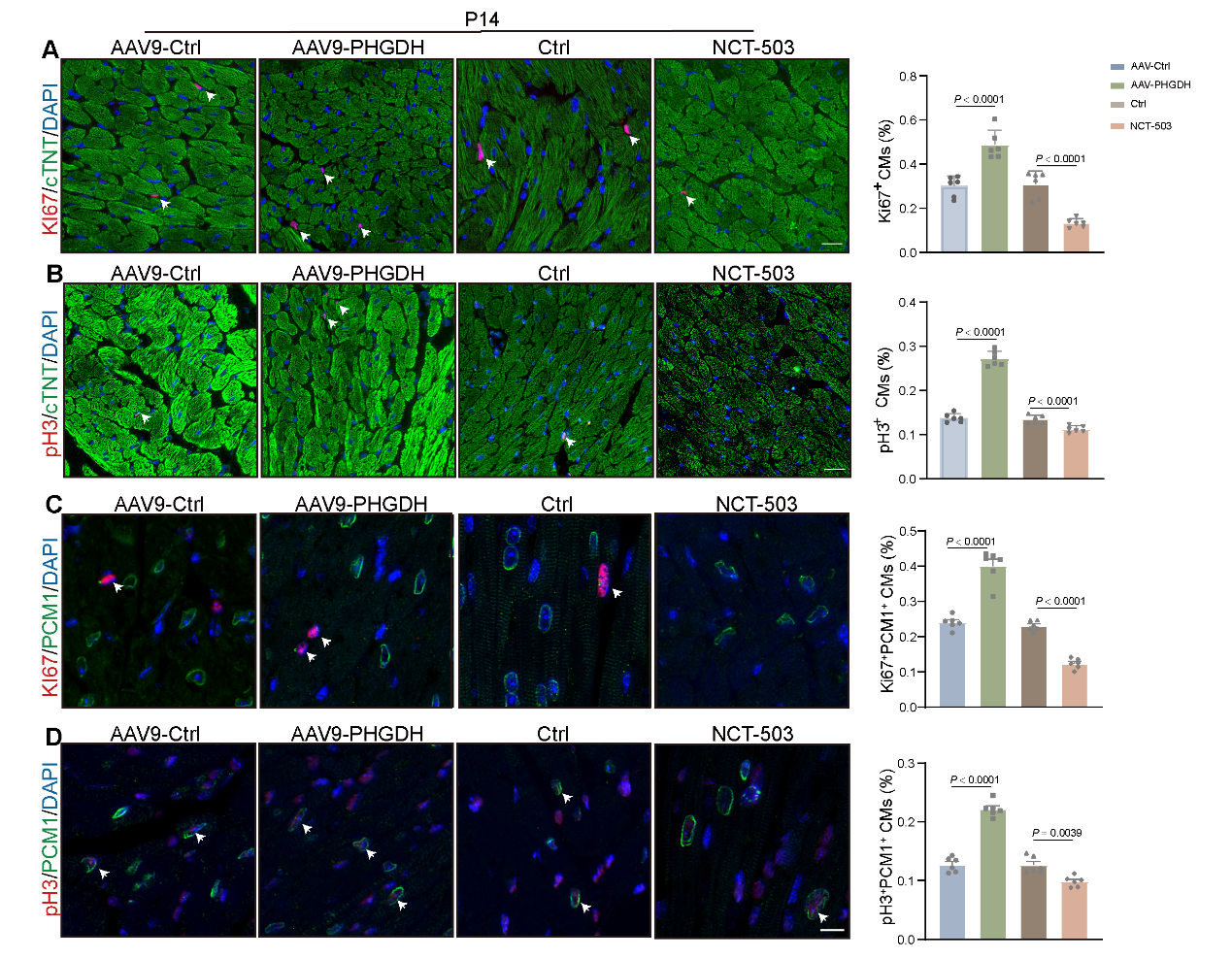


**Figure S3.**

(A and B) Representative immunostaining pictures and statistical analysis of Ki67+ or pH3+ (red) cardiomyocyte numbers in mice hearts at P14 treated with AAV9: cTNT-PHGDH or AAV9: cTNT-Ctrl (n=6/group). Scale bars, 20μm. *P* value as determined by one-way ANOVA. Data are expressed as mean ± S.E.M.

(C and D) Immunostaining images and statistical analysis of co-staining of either Ki67^+^ or pH3^+^ (red) and PCM1(green) CMs in mice hearts at P14 treated with AAV9: cTNT-PHGDH or AAV9: cTNT-Ctrl (n=6/group). Scale bars, 10μm. *P* value as determined by one-way ANOVA. Data are expressed as mean ± S.E.M.


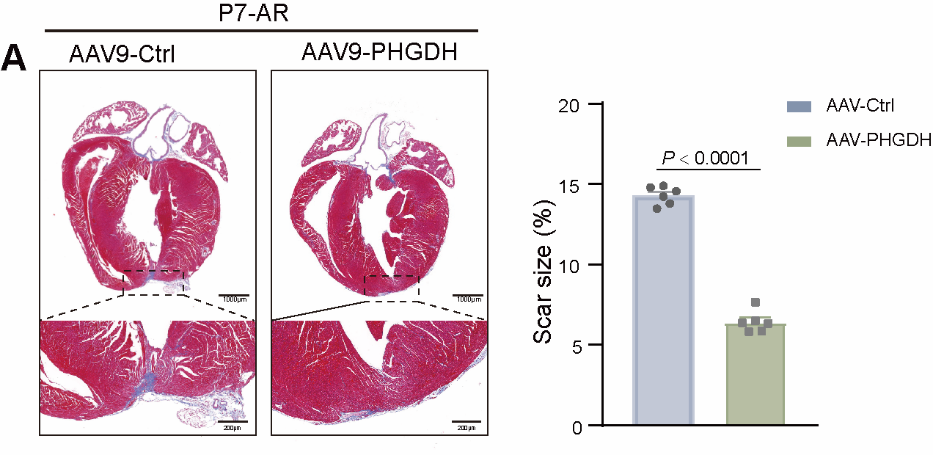


**Figure S4.**

(A) Masson’s trichrome staining for heart regeneration and scar size in P7 mice for apical resection at 21dpr treated with cardiomyocyte-specific AAV9: cTNT-PHGDH compared to AAV9: cTNT-Ctrl (n = 6/group). Scale bars, 1000 μm. *P* value by two-tailed unpaired Student’s t-test. Values were presented as the mean ± S.E.M.


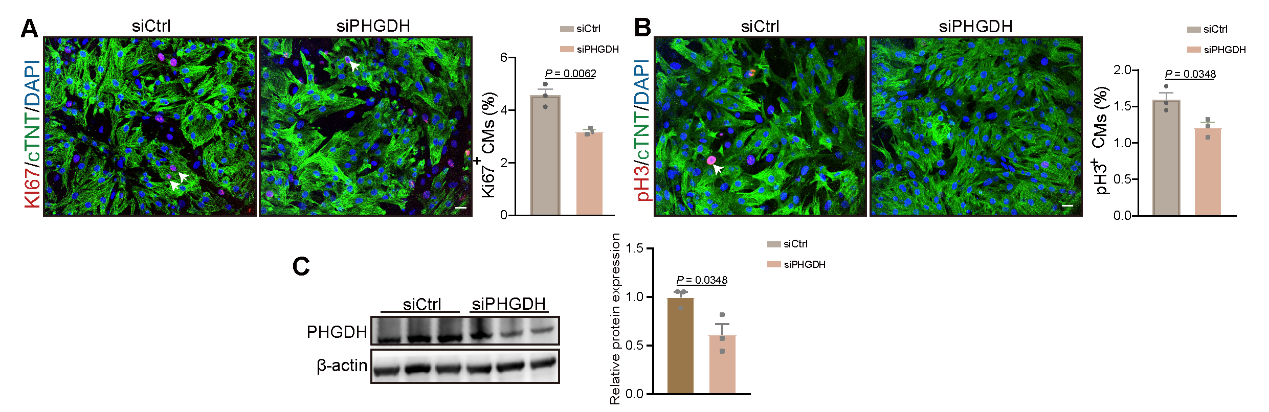


**Figure S5.**

(A and B) Immunostaining images and statistical analysis of Ki67^+^ or pH3^+^ (red) cardiomyocyte numbers in CMs treated with siCtrl or siPHGDH (n=3 per group). Scale bars, 20μm. *P* value as determined by two-tailed unpaired Student’s t-test. Data are expressed as mean ± S.E.M.

(C) Western blot analysis and statistical analysis of PHGDH in CMs treated with siCtrl or siPHGDH (n=3/group). *P* value by two-tailed unpaired Student’s t-test. Values were presented as the mean ± S.E.M.


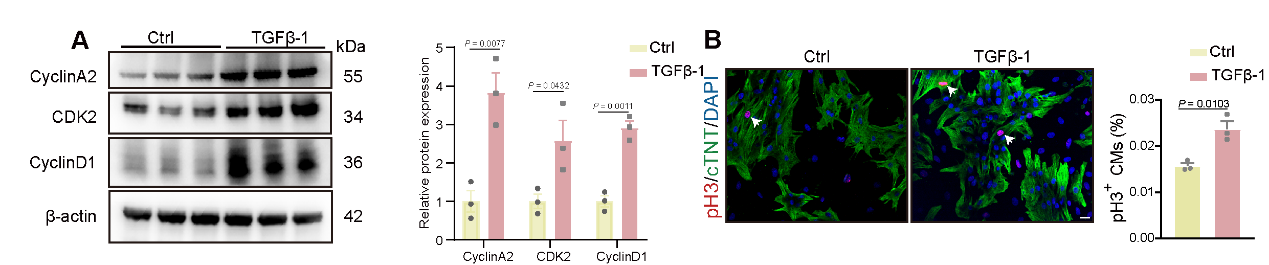


**Figure S6.**

1. Western blot analysis and statistical analysis of CyclinA2, CDK2 and CyclinD1 in CMs

treated with 0.2 ng/mL TGFβ-1 or Ctrl group (n=3, per group). *P* value by two-tailed unpaired Student’s t-test. Values were presented as the mean ± S.E.M.

1. Immunostaining images and statistical analysis of pH3^+^ (red) cardiomyocyte numbers in

CMs treated with 0.2 ng/mL TGFβ-1 or Ctrl group (n=3/group). Scale bars, 20μm. *P* value as determined by two-tailed unpaired Student’s t-test. Data are expressed as mean ± S.E.M.


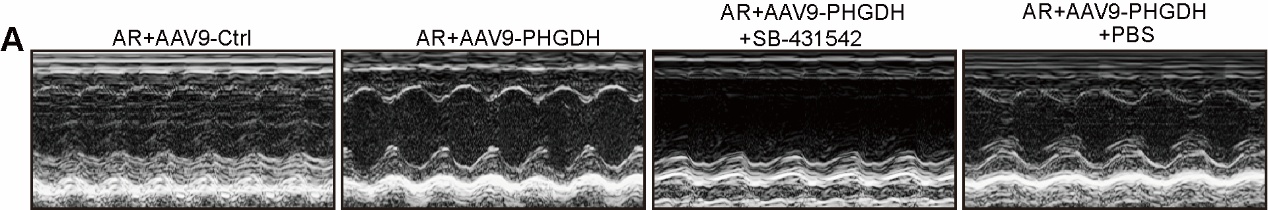


**Figure S7.**

(A) Representative echocardiography images in 21dpr mice treated with AAV9: cTNT-Ctrl, AAV9: cTNT-PHGDH, AAV9: cTNT-PHGDH + 10mg/kg SB-431542 or AAV9: cTNT- PHGDH +PBS (n=6/group)**.**


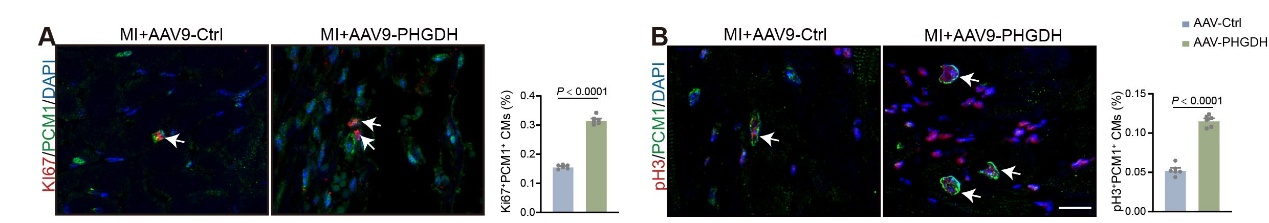


**Figure S8.**

(A and B) Immunostaining images and statistical analysis of co-staining of either Ki67^+^ or pH3^+^ (red) and PCM1(green)CMs in mice hearts at 7 dpi after treatment with AAV9: cTNT-PHGDH or AAV9: cTNT-Ctrl (n = 6/group). Scale bars: 20 µm. *P* value assessed by two-tailed unpaired Student’s t-test. Data are presented as mean ± S.E.M.
